# Psychometric Properties of the Fear of COVID-19 Scale: a Response to Mercado-Lara et al. “Validity and Reliability of the Spanish Version of Fear of COVID-19 Scale in Colombian Physicians”

**DOI:** 10.1007/s11469-021-00635-7

**Published:** 2021-09-13

**Authors:** Chung-Ying Lin, Mark D. Griffiths, Amir H. Pakpour

**Affiliations:** 1 Institute of Allied Health Sciences, College of Medicine, National Cheng Kung University, Tainan, Taiwan; 2 International Gaming Research Unit, Psychology Department, Nottingham Trent University, 50 Shakespeare Street, Nottingham, NG1 4FQ UK; 3 Social Determinants of Health Research Center, Research Institute for Prevention of Non-Communicable Diseases, Qazvin University of Medical Sciences, Shahid Bahounar BLV, 3419759811 Qazvin, Iran; 4 Department of Nursing, School of Health and Welfare, Jönköping University, Jönköping, Sweden

**Keywords:** Fear of COVID-19 Scale, Validity, Reliability, Spanish FCV-19S, Psychometric scale adaptation

## Abstract

A paper reporting the psychometric properties of the Spanish Fear of COVID-19 Scale (FCV-19S) among Colombian physicians was recently published in the *International Journal of Mental Health and Addiction*. Although we welcome the translation and validation of our seven-item scale, this commentary outlines some major concerns we have with the study especially the removal of two items in developing a five-item FCV-19S. Based on these concerns, we strongly recommend that healthcare providers and researchers should use the five-item FCV-19S with caution.

Dear Editors,

Mercado-Lara et al. (2021) recently reported psychometric properties of the Fear of COVID-19 Scale (FCV-19S) in the Spanish language among Colombian physicians in the *International Journal of Mental Health and Addiction.* While the authors’ efforts to use a valid scale to assess individuals’ responses to fear of the coronavirus disease 2019 (COVID-19) pandemic are commendable, we have some major concerns with their study.

1. Although the FCV-19S is in the public domain and can be used freely by anyone (including healthcare providers and other research teams), changes in items or how they are used can jeopardize the assessment in relation to the fear of COVID-19 (Ahorsu et al., 2020; Lin et al., 2021). Unfortunately, Mercado-Lara et al. attempted to develop a brief version of FCV-19S without asking the developers. Although we, the co-developers of the FCV-19S, always welcome interactions and discussions with other researchers for the optimal use of this tool, shortening the FCV-19S without asking us in advance if they could do so was arguably disrespectful and did not follow standard academic etiquette.
2. Solely using the factor analysis results to delete an item is not psychometrically justified. There are many considerations that should be paid attention to when an individual wants to reduce the number of items in a psychometrically robust instrument (i.e., FCV-19S). More specifically, the FCV-19S has been translated and tested in over 20 languages with excellent psychometric properties (Lin et al., 2021). Moreover, the psychometric properties of the Spanish FCV-19S have already been found to be excellent (Huarcaya-Victoria et al., 2020; Martínez-Lorca et al., 2020). Therefore, it is unclear why Mercado-Lara et al. decided to remove the two items from the FCV-19S. One of the highlights for the FCV-19S is the use of questions that examine two types of fear responses (somatic and emotional). During the development stage of the FCV-19S, items were designed to examine both types of fear responses (physical and psychological). Therefore, removing Item 3 (*losing my life*) and Item 7 (*my heart races*) from the FCV-19S in a briefer version of our scale may have the consequence of not capturing individuals’ fear reaction to the COVID-19 pandemic in the way the original scale was developed.
3. Mercado-Lara et al. (2021) developed a Spanish version of the FCV-19S to be used for Colombian physicians. Given that physicians are frontline workers during the COVID-19 pandemic, they may have more extreme experiences of workplace anxiety and fear than those who are not working on the frontline. After reviewing the study by Mercado-Lara et al. (2021), we are unsure whether all of the Colombian physicians surveyed in their study were frontline workers. Therefore, they may have not considered the two deleted items (Item 3 and Item 7) important because they had low contact with COVID-19 patients.
4. Mercado-Lara et al. did not explicitly describe how they culturally adapted the English version of the FCV-19S into the Spanish version. Mercado-Lara et al. mentioned that they utilized the procedure by Guimaraes et al. (2017) for the translation process. However, the study by Guimaraes et al. is not an international guideline for scale translation with cultural adaptation. More specifically, Guimaraes et al. (2017) used the cultural adaptation steps from the guidelines proposed by Guillemin et al. (1993). According to the guidelines proposed by Guillemin et al., (1993) “*a committee should be constituted in order to produce a final version of the modified measure based on the various translations and back-translations*” (p. 1422). Given that Mercado-Lara et al. did not include a committee review in their translation procedure, it is unclear how the minor divergences were resolved in their translation of the FCV-19S into Spanish.
5. Mercado-Lara et al. (2021) did not clearly state how they conducted their confirmatory factor analysis (CFA) (Li, 2016). Because the FCV-19S uses an ordinal scale for the responses, the method of estimation (e.g., diagonally weighted least squares) should be used in the CFA to reduce bias in estimating the factor loadings.

Based on these aforementioned concerns, we strongly recommend that healthcare providers and researchers should use the five-item FCV-19S with caution.
